# Students’ medical ethics rounds: a combinatorial program for medical ethics education

**Published:** 2016-05-01

**Authors:** Maani Beigy, Ghasem Pishgahi, Fateme Moghaddas, Nastaran Maghbouli, Kamran Shirbache, Fariba Asghari, Navid Abolfat-h Zadeh

**Affiliations:** 1 MD-MPH Student, Medical Ethics Association, Students’ Scientific Research Center (SSRC), Tehran University of Medical Sciences, Tehran, Iran;; 2 MD Student, Medical Ethics Association, Students’ Scientific Research Center (SSRC), Tehran University of Medical Sciences, Tehran, Iran;; 3 Associate Professor, Medical Ethics and History of Medicine Research Center, Tehran University of Medical Sciences, Tehran, Iran;; 4 Resident of Ophthalmology, Eye Research Center, Rassoul Akram Hospital, Iran University of Medical Sciences, Tehran, Iran.

**Keywords:** Medical ethics, Medical education, Teaching rounds, Role playing, Large group discussion

## Abstract

It has long been a common goal for both medical educators and ethicists to develop effective methods or programs for medical ethics education. The current lecture-based courses of medical ethics programs in medical schools are demonstrated as insufficient models for training “good doctors’’.

In this study, we introduce an innovative program for medical ethics education in an extra-curricular student-based design named Students’ Medical Ethics Rounds (SMER). In SMER, a combination of educational methods, including theater-based case presentation, large group discussion, expert opinions, role playing and role modeling were employed. The pretest-posttest experimental design was used to assess the impact of interventions on the participants’ knowledge and attitude regarding selected ethical topics.

A total of 335 students participated in this study and 86.57% of them filled the pretest and posttest forms. We observed significant improvements in the knowledge (*P* < 0.0500) and attitude (*P* < 0.0001) of participants. Interestingly, 89.8% of participants declared that their confidence regarding how to deal with the ethical problems outlined in the sessions was increased. All of the applied educational methods were reported as helpful.

We found that SMER might be an effective method of teaching medical ethics. We highly recommend the investigation of the advantages of SMER in larger studies and interdisciplinary settings.

## Introduction

Medical ethics is an important part of the medical curriculum today (1). Presently, all medical schools increasingly require that students be well educated in ethical issues, so as to be equipped with the necessary skills for better management of ethical dilemmas (1-3). It is well recognized that there is no single, best model for medical ethics education; therefore, there was a trend toward developing high quality undergraduate curricula in the past decades (4, 5). The aims of medical ethics education is well portrayed in literature. However, the effective methods of teaching ethics to students have not yet been investigated comprehensively and there is still significant debate on learning and teaching methods (1). It is clear that the current curricular educational methods cannot provide a suitable context for ethical issues to form students’ professional attitudes, because of the different perspectives of medical ethics to the other components of medical knowledge. Medical ethics educators believe the current single, separate course of medical ethics presented during the medical curriculum is insufficient to meet the goals of medical ethics education (1, 6, 7).

Evidence shows medical students and residents have great interest in diverse ethics topics and learning practical skills of preparation for ethical decision-making in clinical situations (5, 8, 9). Moreover, recent recommendations for medical ethics education support the student-centered education in medical curricula (1, 10). The active involvement of students in the process of medical ethics education is advocated (11). In this regard, small group discussion (12-14), problem-based learning (3, 13, 14), case-based discussion (5, 15, 16), ethics grand ward round, ward rounds with ethicists, simulated patients and retreats (1, 7, 17-19), and some other educational methods have been introduced in literature. 

However, there is a lack of information regarding the efficacy of combinatorial programs using the diverse proposed medical education methods. Thus, we conducted a student-based extra-curricular program of medical ethics teaching, and investigated its impact on the students’ attitude and knowledge regarding medical ethics.

## Methods

*Setting and participants*

**Figure 1:**
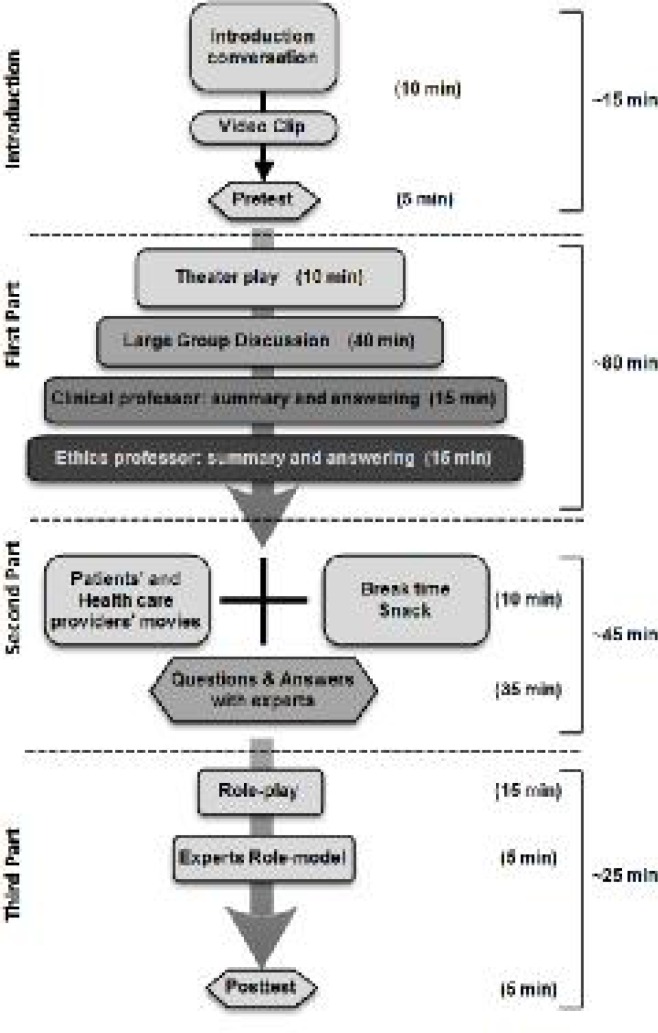
Summary of students’ medical ethics round (SMER) plan

The project of Students’ Medical Ethics Rounds (SMER) was conducted in the Students’ Scientific Research Center (SSRC) of Tehran University of Medical Sciences, Iran, from October 2012 to February 2014 as an extra-curricular program of medical ethics education. All students of medical sciences including medicine, dentistry, nursing, pharmacy, and etc. were eligible and allowed to voluntarily participate in round sessions. The program was designed based on a combination of educational techniques. For assessment of interventions, we used a pretest-posttest questionnaire-based design to evaluate knowledge and attitude changes. Students were informed about the program via letters sent through the Medical Ethics Association (MEA) email list of members, Tehran University of Medical Sciences and SSRC websites, and a few posters in hospitals and departments. A pilot session was held and was followed by 5 other sessions. Each session lasted approximately 3 hours. A summary of each SMER session activities is displayed in figure 1. 

*Project design*

We reviewed the existing literature on medical education methods in the field of medical ethics. We investigated the current methods of medical ethics education in Iranian medical schools including Tehran University of Medical Sciences, and then, we adopted the most effective and feasible methods. We decided to use the peer large group discussion, multimedia, theater-based case presentation, and role play methods. We chose the topics of the sessions based on previous studies on learners’ needs, especially the research conducted in Tehran University of Medical Sciences (20). The pilot study was focused on confidentiality and honesty. The topics of the next 5 SMERs included medical team errors, informed consent, medical education ethics, conflicts of interest, and end-of-life issues, respectively.

*Pilot session*

In order to resolve any unexpected problems, we ran the first session as the pilot session. In the pilot session we reviewed the strength and weaknesses of our methods using participants’ comments. In addition, feedbacks were taken from expert teachers of the round. Based on this small survey, we found priorities regarding the ethical topics to be discussed and educational interventions. 

*Continuous monitoring*

After SMERs, we had a number of meetings for assessment of the rounds. We invited experienced students in the medical ethics and medical education field to the brain-storming session, and discussed the process of the round titled: "How can we improve the effectiveness of SMER sessions?" We used this approach to attaining feedback from the audience and experts throughout the whole project, so that we could observe its encouraging outcomes in the quality of sessions. More exactly, several interventions were added to the SMERs plan based on the aforementioned feedbacks as the rounds were progressing. Moreover, we filmed the round sessions and took pictures of the sessions, so that we were able to review the whole process later, which helped us to be better informed of our performance.

*Medical education interventions*

We used several medical education strategies to perform SMERs. Our rounds were established in the frame of large group discussion in which we used a theatrical play to introduce the ethical dilemma. Video presentation, role playing, and role modeling were the other strategies that were utilized. Large group discussion was conducted as the major part of SMERs. In fact we provided a comfortable environment where students could examine and present their pre-existing knowledge and beliefs, and challenge others’ ideas in a safe environment.

*Patients’ story (scenario) and theater*

The first step of having a theatrical play was writing a scenario to show ethical distress. We chose genuine stories from patients in our hospitals to assure the participants that these events are not limited to ethical books and may also happen to them. Of course, we changed the names of characters and places to respect confidentiality. Every scenario should have 3 parts; exposition, complication, and resolution. However, our scenarios consisted of 2 parts; exposition, through which we introduced the characters and the event, and complication, in which we brought up the ethical challenge. We terminated the play just after the climax, when the ethical dilemma had occurred. Hence, this provided an opportunity for discussing and resolving the dilemma under the supervision of experts in the remainder of the session. It was important for the director to find appropriate actors for the roles given in the scenario. After receiving the script, the first step was to select actors for the play through matching some physical or emotional characteristics of actors and characters of the script. Actors were chosen from the 3^rd^ or 4^th^ year medical students who volunteered and were interested in exploring the enjoyments of the theater. Our preference was to choose students with previous experience in theater. About 3 to 4 rehearsals (according to the complexity of the scenario) were conducted to prepare actors for the performance.

*Experts’ opinions*

We tried to use the experts' opinions after the students' discussion. We invited 2 faculty members; a medical ethics professor to instruct the idealism of ethics, and a clinical professor with ethics teaching experiences to explain the realism of ethics in clinical situations. Furthermore, some other faculty members voluntarily participated in round sessions and expressed their opinions, and thus, they helped us to have a more effective discussion. After students’ group discussion, the expert panel answered the learners’ questions and explained their own ideas and comments about the session’s topic and parts, such as the play, patients' movies, and etcetera. The other roles of the experts in the SMER sessions included summarizing the points of participants’ discussions and explaining specific questions.

*Patients’ films*

To inform the students of the opinions, feeling, and requests of patients regarding certain ethical issues, we made a number of films of real patients. We approached a few of them and asked them to express their opinions while we were filming. For some SMER sessions, we asked health care providers (nurses, students, and etcetera) working in hospitals affiliated to Tehran University of Medical Sciences to express their opinion in front of the camera. Informed consents were obtained orally and we reassured participants during the filming that the films would only be used in SMER sessions. In addition, the wards and hospitals they were admitted to and their personal and medical information were not revealed.

*Pamphlets and guidelines*

At the end of the sessions, we distributed pamphlets containing the most important points discussed. It was used to help participants to remember the general concept of the ethical duties in the discussed ethical issue. In some rounds, we designed algorithms or guidelines in which the suitable approaches to those problems were provided. For developing the pamphlets, we used the most relevant articles and textbooks. Moreover, we presented them to an expert for further editing and revision. We used the instructions provided in the Writing Effective Pamphlets guideline by Newell (21).

*Role playing and role modeling*

At the end of each SMER session, we performed a short modified format of role playing. We presented a problematic or dilemmatic scenario and asked the participants to imagine that they are the person involved in those situations and act out what they would have done. In this way, they have the opportunity to practice what they have learned. Finally, we asked 1 expert (usually the clinician) to act out the same situation as an active role-model for learners. 

*Tests and statistical analysis*

Pretest and posttest were designed under the supervision of experts of the main SMER topics. The items of the questionnaires were extracted from the best articles and textbooks. The questions were in the form of statements to which the respondents gave their answer through a 5-point Likert scale including strongly agree, agree, neither, strongly disagree, or disagree. We evaluated the face validity of the questionnaires by obtaining expert feedback on the questionnaires. The internal consistency reliability of questionnaires was analyzed using Cronbach’s alpha. Data are expressed as numbers (percentages) for certain responses. For convenience, we transformed the 5-point Likert scale to a binomial scale (true or false, bad or perfect, and etcetera) to better describe the participants’ responses in the tables. Differences between pretest and posttest responses were analyzed using the Wilcoxon signed-rank test. Between items, differences were analyzed using the Mann-Whitney U test. Data were analyzed using SPSS software (version 16, SPSS Inc., Chicago, IL, USA). The *P*-value of less than 0.05 was considered statistically significant.

## Results

A total of 335 students participated in this study and 85.6% of them (290 students) filled the pretest and posttest forms. The average age (mean ± SD) of participants was 22.22 ± 2.3, the majority of participants were female (207 girls, 71.5%), and 94.5% (274 students) were medical students. In addition, 81% of individuals were in their first 4 years of education; thus, they had not passed the ethical course of the medical curriculum. Moreover, students were not obliged to participate in all sessions. Participants’ opinions on each subject of SMER are summarized in table 1. In the total of rounds, 89.2% of participants reported that the theater group could successfully display the ethical problem. The presenter’s (i.e., lecturer’s) role of facilitation of discussion was described by 96.7% of participants as a perfect performance. Large group discussion was reported to be effective by 88.3% of participants. Approximately 87% of audiences were satisfied with the professors’ role in answering the participants’ questions and summarizing the important points in each session. Nearly 90% of audiences thought the subjects of sessions were practical and necessary. Interestingly, 89.8% of participants reported that their confidence regarding how to deal with the ethical problems outlined in the session had increased. Role-play was reported as an effective method by 80.9% of students, and 86.3% of participants requested that pamphlets be provided for them.

**Table 1:** Participants’ reflections on the SMER plan

**Items** *	**Participants’ responses** #	**Students’ Medical Ethics Rounds topics**						**Difference**
		**Medical errors (%)**	**Informed consent (%)**	**Medical education (%)**	**Conflict of interest (%)**	**End-of-life issues (%)**	**Total of rounds (%)**	***P*** **-value†**
Theater	Bad	11.8	14.3	14.3	6.8	3.4	10.8	0.249
	Perfect	88.2	85.7	85.7	93.2	96.6	89.2	
Presentation	Bad	0	3.1	2.4	4.5	0.0	3.3	0.013
	Perfect	100	96.9	97.6	95.5	100.0	96.7	
Large group discussion	Bad	2.9	15.6	0	6.8	3.4	11.7	< 0.0001
	Perfect	97.1	84.4	100	93.2	96.6	88.3	
Professors	Bad	0	26.6	14.3	4.5	34.5	12.3	0.010
	Perfect	100	73.4	85.7	95.5	65.5	87.7	
Practicality	Bad	7.9	11.1	19	4.5	0.0	10.2	0.275
	Perfect	92.1	88.9	81	95.5	100.0	89.8	
Confidence	Bad	31.6	25	33.3	25.6	35.7	10.2	0.507
	Perfect	68.4	75	66.7	74.4	64.3	89.8	
Pamphlet	Bad	15.8	Not asked	14.3	9.1	6.9	13.7	0.187
	Perfect	84.2		85.7	90.9	93.1	86.3	
Role-play	Bad	7.9	11.1	19	34.9	10.3	19.1	0.090
	Perfect	92.1	88.9	81	65.1	89.7	80.9	

* These items enquire into the participants’ satisfaction regarding the quality of educational methods used in each SMER.

# The participants’ responses regarding the quality of these items were, first, obtained using a 5-point Likert scale, then, transformed to this binomial scale for convenience.

†For total (bad/perfect) answers, Z-test


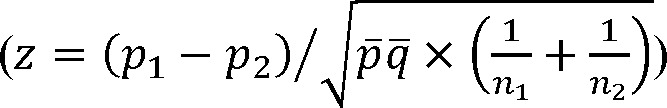


was performed, which was consistent with results of the Wilcoxon signed-rank test for the 5-point Likert scale.

Participants’ responses to the knowledge questionnaires are summarized in table 2. Only 4 knowledge questions were provided in each session. In the total of the SMERs, the students’ knowledge was significantly increased (*P* < 0.05).

Results of participant’s responses to the attitude questionnaires are summarized in table 3. Their responses were transformed from a 5-point Likert scale to the positive and the negative attitudes. Interestingly, we observed that in the total of SMERs, the positive attitude of participants had significantly (P < 0.00001) increased.

**Table 2:** Participants’ responses to knowledge pretest and posttest

**Items** *	**Participants’ responses** #	**Students’ Medical Ethics Rounds topics**					
		**Medical errors (**%**)**	**Informed consent (%)**	**Medical education (%)**	**Conflict of interest (%)**	**End-of-life issues (%)**	**Total of rounds (%)**
Pretest	False	45.45	32.2	42.22	58.3	65.56	45.45
	True	54.55	67.8	57.78	41.7	34.44	54.55
Posttest	False	21.05	25.3	32.5	53.98	38.26	21.05
	True	78.95	74.7	62.5	46.02	61.74	78.95
Difference	*P*-value	**0.0017**	0.1446	0.3129	0.3358	**0.0029**	**< 0.00001**

* These items enquire into the participants’ knowledge regarding the topic of each SMER. English translations of them are provided in supplementary material.

# The participants’ responses were, first, obtained using a 5-point Likert scale, then, transformed to this binomial scale for convenience. In addition, we summed the total responses (true/false) of all knowledge questions.

†For total (true/false) answers, Z-test


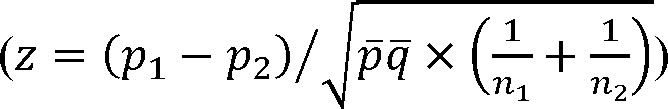


was performed, which was consistent with results of the Wilcoxon signed-rank test for the 5-point Likert scale.

**Table 3:** Participants’ attitude scores in pretest and posttest

**Items** *	**Participants’ attitude** #	**Students’ Medical Ethics Rounds topics**					
		**Medical errors (%)**	**Informed consent (%)**	**Medical education (%)**	**Conflict of interest (%)**	**End-of-life issues (%)**	**Total of rounds (%)**
Pretest	Negative	47.2	18.1	56.3	40.3	64.4	42.3
	Positive	52.8	81.9	43.7	59.7	35.6	57.7
Posttest	Negative	28.1	11	43.6	10.9	47.1	25.4
	Positive	71.9	89	56.4	89.1	52.9	74.6
Difference	*P*-value**†**	**< 0.00001**	0.08	0.143	**< 0.00001**	**< 0.00001**	**< 0.00001**

* These items enquire into the participants’ attitude regarding the topic of each SMER. English translations of them are provided in supplementary material.

# The participants’ responses were, first, obtained using a 5-point Likert scale, then, transformed to this binomial scale for convenience. In addition, we summed the total responses (true/false) of all knowledge questions.

†For total (true/false) answers, Z-test


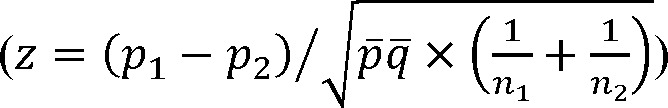


was performed, which was consistent with results of the Wilcoxon signed-rank test for the 5-point Likert scale.

## Discussion

In the traditional model of medical ethics education, medical ethics is taught as a separate course during the clinical years of the undergraduate medical curricula in Iranian medical schools. However, incorporating these ethical principles into clinical training still remains challenging (22). Furthermore, lecture-based education has been demonstrated to be insufficient in terms of empowering students to employ their knowledge in clinical reasoning (1, 2, 6, 7). There is increasing evidence supporting methods in which students are more involved in the learning process including ethics grand ward rounds, ward rounds with ethicists, and simulated patients and retreats (1, 3, 7, 17-19). Moreover, literature supports the advantages of innovative student-based programs in which students watch each other role play and discuss clinical tasks, such as obtaining informed consent, giving bad news, and discussing do not resuscitate orders (3, 7, 23, 24). Thus, we designed an innovative extra-curricular program of teaching medical ethics in a student-based project, in which a combination of educational methods were employed. 

We found that this program could successfully attract medical students of Tehran University of Medical Sciences and could satisfy their expectations of an open environment for discussing ethical dilemmas. Fortunately, the results of pretest and posttest showed a significant increase in self-confidence, knowledge, and attitude scores in the total of rounds. Almost all of the educational methods were considered helpful by the participants. There were no extracurricular, student-based, combinatorial programs of medical ethics education in the literature; there were few, if any, extracurricular studies which were confined by single methods. However, here we provide the most similar experiences of medical ethics education, and try to compare their effectiveness. The study by Parker et al. which is the most similar intervention to our study includes student case presentations during a modified teaching ward round model named "clinical ethics ward rounds" (7). Interestingly, the cases presented by students in "clinical ethics ward rounds" were very similar to the subjects of SMER; as unethical behavior in others, confidentiality, end-of-life issues, autonomy, and equity were the most common presented cases (7). The importance of peer discussions in maturation of ethical thinking, which is well elucidated (25, 26), has been tried to be covered both in SMER and "clinical ethics ward rounds" (7) through the forums provided by these studies. Another similar study was conducted by Fryer-Edwards et al. employing "Ward Ethics Sessions" (22) including peer discussions supported by mentors and faculty members. Both "clinical ethics ward rounds" and "Ward Ethics Sessions" showed that engagement of students in discussions and their confidence in encountering ethical dilemmas were improved, similar to our findings. Another recent effort to compare problem-based learning and small group discussion methods by Heidari et al. (14) showed mild non-significant higher scores of problem-based learning compared to small group discussion. Our results regarding the importance of involvement of students in medical ethics education are also in line with the study by Huijer et al. (25). They concluded that we should encourage students to express their opinions and deal with values, responsibilities, and the uncertainty and shortcomings of medical interventions (25). Similar to our cases, issues of informed consent, end-of-life decisions, and medical errors were the most common presented cases by students in this study (25).

One may inquire into how "ethics rounds" can be effective in medical ethics education. The round-based method [various names have been used, e.g., ward ethics sessions (22), clinical ethics ward rounds (7), and clinical rounds in medical ethics (18)] for ethics education was employed for the first time several years ago. To the best of our knowledge, clinical rounds in medical ethics were established, for the first time, in 1971 in a large medical center (Children's Hospital Medical Center in Boston, USA) (18). This method was used to provide a forum for the multidisciplinary discussion of moral dilemmas in health care with the aim of "continuing education" in medical ethics (18). Their rounds included case presentations and interpretations provided by interdisciplinary discussions of law, pediatrics, religion, and philosophy professionals (18). Thereafter, this format was employed in several hospitals and medical universities with the aims of improving decision-making in medical staff and continuous education of professionals. Several years later, after the introduction of more powerful educational methods in literature (e.g., problem-based learning), some scholars tried to use "ethics rounds" method for ethics education of undergraduate medical students and interns. Fryer-Edwards et al. tried to incorporate ethics education into the clinical years through employing "ward ethics sessions" at the University of Washington (22). Through supported peer discussions (supervised by mentors or faculty members) of ethical issues, students were allowed to develop their own moral compass and intuition regarding appropriate training behaviors and practices (22). Moreover, their ability to identify issues, develop responses to ethical distresses, recognize their own responsibility, and identify necessary skills for appropriate actions was improved (22). Somewhat similar successes were reported by Parker et al. (7) regarding the use of the "round method", as mentioned above. The feedbacks we obtained from students and faculty members participating in SMERs were very similar to the study of Fryer-Edwards et al. (22); that clinical years [it has been referred to as "clinical clerkship and internship" in Tehran University of Medical Sciences (27)] are a fruitful period to shape professionalism and ethics. As it has been previously elucidated regarding hidden curriculum (6, 28), students observe, learn, and imitate the behaviors and interaction styles of doctors with peers, patients, and staff (22, 29, 30). The other important feedback we received was the isolation of students in clinical environment which resulted in them rarely finding the opportunity to discuss many of these ethical distresses with peers. This finding was also similar to that of the study by Fryer-Edwards et al. (22). It is clear that without exclusive forums for supported peer discussions on ethical dilemmas, medical students undergo "ethical erosion" ["a phenomenon of decreased ability to recognize and respond appropriately to ethically problematic behavior" (22)] that has been previously elucidated in literature (31). In addition, the enthusiasm and participation of students in SMER was very encouraging for faculty members, especially ethics professors who always reported insufficient interest of students in lecture classes.

It should be noted that professionalism was also considered in the SMER program (especially in sessions of medical education, medical errors, and end-of-life issues). There is a growing body of evidence about various teaching methods of professionalism (as well as ethics) (32, 33). The didactic lecture is the most common and efficient method for "summarizing large amounts of information", which can improve knowledge and change attitudes (32), but has its own imperfections as lectures can rarely change behaviors and performances (34). The plan of our rounds (Figure 1) included a theatrical play at the beginning to introduce the ethical problem. The theatrical play at the beginning can enhance the realization that patients have of "narrative" lives, and that every patient represents a singular event situated within a more complex contextual structure (35). After that, through large group discussion, the majority of participants were actively involved in the discussions and the remaining were active listeners. Then, professors summarized the most important points of the ethical problem and answered the participants’ questions. Furthermore, through the group discussion and using innovative visual material such as theatrical play and patients’ films, we increased participants’ interest in the subject, put the burden of learning on them, and increased learners’ involvement (35). Group discussion also provides learners with the opportunity of immediate feedback and is useful for guiding learners toward higher levels of thinking and inquiry (35). The other advantages of this educational strategy include providing valuable clues about learners’ motivation and how to better facilitate learning and help students to identify and build on preexisting knowledge (35). In patients’ videos, we could portray realistic situations and patients’ opinions (36). It is recommended to employ multimedia to enhance teaching and learning (e.g., movies on professional and unprofessional behaviors and requesting audience responses after presentation of ethical scenarios) (32). Another large group discussion was performed after the video presentation of patients’ opinions and reflections. After the second large group discussion and the professors’ responses and opinions, modified role playing was performed in which some students were requested to voluntarily play in selected ethical problem situations relevant to the SMER topic. Role playing actively involves participants, develops problem-solving and communication skills in learners, and enables learners to experience in a safe environment with behaviors which strike them as potentially useful and to identify the useless ones (35). Recently, use of role playing for undergraduate teaching of ethics was investigated (37). While it showed similar results to that of the lecture method, it seems the students’ satisfaction with and involvement in the education process is increased by role playing (37). Furthermore, role playing provided us immediate feedback about the learner’s understanding and ability to apply concepts. Another successful example of using role playing in the literature is the “Breaking Bad News” course at the London Hospital Medical College and St Bartholomew's (38). They employed group discussion, video presentations, and role play involving actors, to develop students’ skills in “breaking bad news” (38). Role modeling (an expert clinician’s role play in front of participants) was the other strategy used in SMER to introduce professional practice to learners, which helps students to copy appropriate ethical behaviors (1, 32, 35). Role modeling has been demonstrated to be an effective means of teaching professionalism (32). 

In this program, we encountered some limitations. We only ran a few rounds with heterogeneous participants, so we were not able to evaluate the effect of our program on their behavior and their ethical reasoning. The other limitation was the content validity of the knowledge questionnaire. Very few questions were included in the knowledge questionnaire because of the time limitation, so the content validity of this tool might not be ideal. However, the results of questionnaires and feedbacks of participants showed that this program can light the way to a new method of conducting more interesting/effective programs of medical ethics education.

## Conclusion

In summary, for the first time, we introduced an innovative combinatorial medical ethics education program, conducted in an extra-curricular student-based project named Students’ Medical Ethics Rounds (SMER). We employed theater case presentation, large group discussion, expert opinions, role playing, and role modeling methods in SMERs. All of the methods were reported by participants to be advantageous. Furthermore, pretest and posttest results showed us significant improvement in knowledge and attitudes of students. This study represents a research in a local university, but we believe that the results provide new and effective guidance on structuring medical ethics courses for teachers around the world. It seems necessary for future researches on frameworks similar to SMER to consider student involvement in managing or planning actions.

## Notes:

**Practice Points**

•     The current single, separate course of medical ethics during the medical curriculum is insufficient to meet the goals of medical ethics education.

•      Combination of educational methods, like large group discussion, multimedia, theater-based case presentation, and role play, are very effective in teaching medical ethics.

•       Medical students are potential human resources for enhancing effectiveness of educational programs.

•       There is increasing evidence to support student-based teaching of medical ethics.

**Ethics:**

Ethics is the branch of philosophy that deals with distinctions between right and wrong and with the moral consequences of human actions. Examples of ethical issues that arise in medical practice and research include informed consent, confidentiality, respect for human rights, and scientific integrity.

**Questionnaires**

Please determine your level of agreement to the statements below in terms of strongly agree, agree, neither, disagree, or strongly disagree.

A) Participants’ reflections on the Students’ Medical Ethics Rounds (SMER) plan

1. Theater group could successfully reflect the ethical problem of this session.

2. The presenter persuaded all participants in the discussion well.

3. The group discussion was not conducted appropriately (i.e., there was no difference between this session and university lectures).

4. Professors provided a useful summary and responded well to the participants' questions.

5. The subject of this session was practical and useful to me.

6. My confidence has been improved regarding how to encounter the discussed ethical situation after this session.

7. The provided pamphlet was useful for learning the ethical subjects.

8. Role playing helped me to improve my skills for a more ethical performance.

B) Knowledge and attitude questions

1. When I make a mistake during health care services, I do not disclose it to patients and their relatives, mainly in order to not ruin their beliefs in the medical team.

2. When I witness my colleague's mistake (with possible harms to patients), I directly disclose it to patients.

3. I think disclosing errors to my patient damages the physician-patient relationship.

4. In cases of medical errors with minor damages, patients must not be informed about them.

5. All medical errors should be judged based on the severity of the damage; thus, only important harms must be disclosed.

6. Disclosure of medical errors lightens the patient-physician relationship and improves the health care service.

7. Once my superiors make medical errors, I know what to do.

8. I am self-confident and skillful enough to disclose my own medical errors to patients.

9. Patients prefer not to know health care providers' errors, because it disturbs them.

10. I believe it is unethical not to disclose my own medical errors.

11. I am able to convince my colleagues to disclose their medical errors to their patients when necessary.

12. I am usually reluctant to disclose my errors, in spite of guilty feelings.

13. I know what to do when either me or my colleagues make medical errors.

14. I am worried to be considered as a non-professional when disclosing my colleagues' error to somebody else.

15. Disclosing medical errors increases the medical charges.

16. Disclosing my own medical errors to my patients disrupts my relationship with them.

17. Informed consent is a practical subject of medical ethics, and it is necessary to acquire skills for it.

18. I am confident enough to manage the cases of deciding about preparing written informed consent.

19. It is the patients' right to give their written informed consent before procedures.

20. In emergent and threatening conditions, the obtaining of an informed consent can be neglected.

21. It is not necessary to obtain written informed consent from patients with mental disorders.

22. It is not necessary to obtain written informed consent from patients under the age of 18 years.

23. I know ethical considerations of medical education, acknowledgement of which are necessary during my clinical education courses.

24. I always introduce myself as a medical student to patients.

25. I always do my best to cause minimal harm but provide maximal benefit to patients.

26. If the patient is reluctant to tell me her/his history, I will not try to force her/him by asking the professors.

27. Practicing procedures on newly dead patients is correct when professors permit.

28. For involvement of patients in clinical education, informed consent should be obtained.

29. Actions during clinical education of students must be advantageous for patients.

30. Performing procedures is against the ethical law of nonmaleficence, because of the possibility of damages.

31. Patients' satisfaction decreases during clinical education of patients.

32. Conflict of interests is a practical subject of medical ethics, and it is necessary to acquire skills for it.

33. I am able to recognize any conflict between my interests (as the physician) and my patients' interests.

34. I know how to decide in cases of conflict of interests.

35. Physicians should never accept gifts from pharmacy manufacturers.

36. Physicians should not directly accept funds for travelling (or registration) for scientific congress from pharmacy manufacturers.

37. Advertising medical instruments or medications by health care providers in their offices is forbidden, based on local (Iranian) regulations.

38. Inviting or attracting patients from governmental medical centers to private clinics is forbidden, based on Iranian regulations.

39. Patients' rights should never been neglected by medical students because of justifications like having exams and etcetera.

40. I have enough knowledge about medical ethics considerations of end-of-life care.

41. I believe the physician is allowed to fulfill the patients' will for terminating his life (euthanasia).

42. Communication with terminal patients about their prognosis damages them.

43. Due to Iranian regulations, cessation of life-maintaining interventions for terminal patients is forbidden even for requesters.

44. The request of patients' relatives regarding life-maintaining interventions for terminal patients does not have a role in physicians’ decision-making.

45. I have enough skills regarding communication with terminal patients about death and end-of-life issues.

46. I believe it is good to perform fake cardiopulmonary resuscitation for terminal patients (less than necessary time of protocols) to satisfy their relatives.
